# THE ASSOCIATIONS OF GRIP STRENGTH WITH CO-RESIDENCE WITH ADULT CHILDREN AND MORTALITY CHINESE OLDER ADULTS

**DOI:** 10.1093/geroni/igad104.2252

**Published:** 2023-12-21

## Abstract

Aging in place is a key to coping with population aging in China. Grip strength (GS) is one of the key determinants of aging in place. Under such a context, whether living with adult children would affect older adults’ GS and mortality is worth exploring. The objective of this study was to assess the associations of GS with adult children co-residence and mortality, and examine whether the association between GS and mortality would be moderated by adult children co-residence status. We used a nationally representative sample of Chinese older adults who completed three-wave (2011, 2013, and 2015 years) of the China Health and Retirement Longitudinal Study (N=1,088, aged 50-100 years). Mortality status was determined by in-person interviews in waves 2 and 3. GS was treated as a continuous variable in mixed-effect models and a binary variable in Cox regression models (poor GS if GS< 18kg for women and GS< 28kg for men). On average, 52.2% (n=535) participants co-resided with adult children. Adult-children co-residence was longitudinally related to better GS over time (β=2.96, 95% CI=0.53-3.60, p=0.009). Overall, poor GS was an independent risk factor for mortality (HR=1.33, 95% CI=1.04-1.70, p=0.022) after controlling for covariates (e.g., age, sex), but this association was not modified by co-residence status (HR=1.02, 95% CI=0.64-1.64, p=0.900). These results suggest that co-residence with adult children might be beneficial to better GS, which might contribute to less mortality. Future studies are needed to explore the underlying mechanisms through which the co-residence affects GS, thus, contributes to mortality.

